# 

**Published:** 1867-01

**Authors:** 


### NEW BOOKS.

We have received the following new publications from the enterprising Publishing House of Henry C. Lea:

*A Treatise on Principles and Practice of Medicine, designed for the use of Practitioners and Students of Medicine.* By Austin Flint, M. D., Professor of the Principles and Practice of Medicine in the Bellevue Hospital Medical College, and in the Long Island College Hospital: Fellow of the New York Academy of Medicine, &c. Second Edition revised and enlarged.

This work has been before the profession but little more than a year, and has already reached a second edition. It evinces much patient labor, and, in its style, meets our views of such a work. The reputation of Dr. Flint guarantees to the profession a reliable standard authority, and as such we commend it to public favor.

*A Practical Treatise on Fractures and Dislocations.* By FRANK HASTINGS HAMILTON, A. B., A. M., M. D.

Professor of the principles of Surgery, Military Surgery and Hygene, and of fractures and dislocations in Bellevue Hospital Medical College: Surgeon to Bellevue Hospital and to the Charity Hospital, New York: Professor of Military Surgery, &c., in the Long Island College Hospital: Author of a Treatise on Military Surgery. This Edition revised and improved—Illustrated with two hundred and ninety-four wood cuts.

We have not had time to peruse this work. We will do so and give our opinion of it. Its mechanical execution is very fine, and the wood cuts are equal to any we ever saw.

